# Cognitive behavior therapy for diabetes distress, depression, health anxiety, quality of life and treatment adherence among patients with type-II diabetes mellitus: a randomized control trial

**DOI:** 10.1186/s12888-023-04546-w

**Published:** 2023-02-03

**Authors:** Qasir Abbas, Sana Latif, Hina Ayaz Habib, Salman Shahzad, Uzma Sarwar, Mafia Shahzadi, Zoobia Ramzan, Washdev Washdev

**Affiliations:** 1 Department of Applied Psychology, Government College University Faisalabad, Old Campus, Faisalabad, Pakistan; 2 Institute of Clinical Psychology, University of Karachi, Karachi, Pakistan; 3 Department of Psychology, Government College Women University Sialkot, Sialkot, Pakistan; 4 Department of Applied Psychology, Government College University Faisalabad, Main Campus, Faisalabad, Pakistan; 5 Institute of Behavioral Sciences, Dow University of Health Sciences, Karachi, Pakistan

**Keywords:** Cognitive behavior therapy, Diabetes distress, Depression, Health anxiety, Treatment adherence, Type 2 diabetes mellitus

## Abstract

**Objective:**

Diabetes distress typically causes depressive symptoms; common comorbidity of diabetes unpleasantly affects patients’ medical and psychological functions. Psychotherapeutic interventions are effective treatments to treat depressive symptoms and to improve the quality of life in many chronic diseases including diabetes. The present study investigated the efficacy of cognitive behavior therapy (CBT) to treat depressive symptoms in patients with type 2 diabetes mellitus (T2DM) using experimental and waitlist control conditions.

**Materials and Methods:**

A total of 130 diagnosed patients with T2DM were taken from outdoor patients services of different hospitals in Faisalabad. Ninety patients met the eligibility criteria and were randomly assigned to experimental (*n* = 45) and waitlist control (n = 45) conditions. All the patients completed clinical interviews and assessment measures at pre-and post-assessment stages (16 weeks intervals). Medical consultants at the respective hospitals diagnosed the patients on the base of their medical reports and then referred those patients to us. Then we used different scales to assess primary and secondary outcomes: Diabetes Distress Scale (DDS) and Patient Health Questionnaire (PHQ) to assess primary outcomes, and a Short Health Anxiety Inventory (SHAI), a Revised Version of the Diabetes Quality of Life Questionnaire (DQLQ), and a General Medication Adherence Scale (GMAS) were used to investigate secondary outcomes. Repeated measure ANOVA was used to analyze the results.

**Results:**

The findings indicated that patients who received CBT got a significant reduction in their diabetes distress F(1,60) = 222.710, *P* < 0.001, η^2^ = .788), depressive symptoms F(1,60) = 94.436, *P* < 0.001, η^2^ = .611), health anxiety F(1,60) = 201.915, *P < .*0.001, η^2^ = 771), and a significant improvement in their quality of life F(1,60) = 83.352, *P <* 0.001, η^2^ = .581), treatment adherence F(1,60) = 67.579, *P <* 0.001, η^2^ = .566) and physical activity schedule F(1,60) = 164.245, P < .0.001, η^2^ = .736 as compared to the patients in waitlist control condition.

**Conclusion:**

It is concluded that cognitive behavior therapy is an effective and promising intervention for depressive symptoms, diabetes distress, and health anxiety which also helps the person to promote quality of life, treatment adherence and physical activity.

**Supplementary Information:**

The online version contains supplementary material available at 10.1186/s12888-023-04546-w.

## Introduction

Diabetes or diabetes mellitus is a metabolic disease that interferes with the human body’s ability to process and absorb glucose. It is the seventh leading cause of death, and about 422 million people live with diabetes worldwide (WHO 2020). The current scenario estimates that it will rise by 25% within 10 years to 454 million and 51% within 25 years to 548 million [1]. In Pakistan, the prevalence of type 2 diabetes is 13.7% higher in urban areas [2]. According to National Diabetes Federation (2019), about 19 million in Pakistan from age 20 to 79 have been suffering from diabetes. Type-II diabetes is the most commonly occurring type that accounts for up to 90% of the total [3].

Diabetes could be a reason of a variety of psychological disturbances in the people suffering from it. One such psychological disturbance is called diabetes distress (DD). DD is a big problem that accompanies emotional disturbances, stress, guilt feelings, and avoidance of treatment [4]. It is more frequent among patients with type II diabetes mellitus [5]. The prevalence of DD globally is around 45% which is quite high as it is a predictor of clinical outcomes among patients of T2DM [6] Poor self-care, poor self-management, and a reduced treatment adherence among patients with diabetes are some of the negative treatment outcomes associated with DD [7]. Depression is a mental state characterized by a pessimistic sense of inadequacy and lack of activity, usually occurring a i when the intensity of distress becomes high as frequent and unmanaged distress can sometimes lead to full-blown depression [8]. Many factors can lead to depression such as psychological, social, and biological and it can also present itself as a comorbid condition of chronic medical illnesses such as diabetes, [9]. Having a depressive disorder increases the risk of new-onset diabetes mellitus, and patients with diabetes mellitus also have a higher likelihood of developing depressive symptoms. Therefore, depression is an observed common factor among diabetic patients and it frequently co-occurs [10].

Health anxiety (HA) is another factor which causes distress; it occurs when an individual misinterprets his bodily sensations or changes as the indicators of a life-threatening illness (Asmundson & Taylor, [11]). HA is the core of many psychological illnesses, ranging from low to severe [7]. DD leads to HA because patients with chronic illnesses such as diabetes commonly experience fears of illness or symptoms recurring or worsening. It leads to lower adherence to treatment, fewer positive health behaviors, and increased medical costs [12]. HA is higher among patients with diabetes [13].

Treatment adherence is usually described as the patient’s compliance with the prescribed medication [14]. Treatment adherence is necessary to control diabetes and to foreclose death rate and severe morbidity. Adherence to treatment in diabetes helps maintain proper health and reduces diabetes-related complications. Albeit, some psychological factors may compromise compliance and adherence of patients with diabetes mellitus [14]. Quality of life (QOL) refers to how much an individual is healthy, comfortable, and can participate in different events of life or can enjoy life events. It is an important variable to be considered in any healthcare research as it measures life in terms of participation in life not in terms of lived years [15]. Individuals with high quality of life usually have an improved metabolic control of their disease as compared to those with lower quality of life, whereas, patients with higher blood glucose also have a lower health-related quality of life [16].

Cognitive Behavior therapy (CBT) is a form of psychological intervention which emphasizes the current state of affairs and, usually, it is a time-bound mode of therapy [17]. CBT uses the cognitive-behavioral model, which taps the thought patterns of an individual that are triggered by their behavioral and physiological reactions to different stimuli [18]. Cognitive behavior therapy does not merely work for the management of psychiatric disorders, recent decades of research have provided evidence of the effectiveness of CBT with many different chronic illnesses and their related psychological issues; CBT also helps reduce depression and anxiety among patients with diabetes [19, 20].

Recent studies show that, in Asian countries, there is a need for improved mental health screening and treatment in diabetes care (Karla et al., 2020). CBT works effectively with depression and increases treatment adherence in diabetes patients as CBT is an evidence-based treatment therapy for depression as a comorbid condition with diabetes [21].

CBT has the potential to addresses the emotional problems of patients with T2DMas it has been practiced with T2DM to reduce depressive symptoms [22]. CBT has been substantiated to be an effective treatment intervention for diabetes distress [23], reduction of emotional problems [24], improvement in adherence [25] and for control of glucose level through adherence [26].

This current study aimed to examine the effectiveness of CBT through experimental and waitlist control conditions with type 2 diabetes patients. We hypothesized that CBT will effectively reduce diabetes distress and other depressive symptoms, will effectively deal with health-related anxiety concerns and distress due to diabetes, and will improve the patients’ treatment adherence and quality of life.

## Research design and methods

### Study design

The current research is a prospective randomized control trial (RCT) in which we assessed the effectiveness of CBT using EXP and WLC conditions with T2DM patients using pre-and post-test measures. Outcome measures were obtained at the baseline and post-interventions. The participants were taken from different public and private hospitals of the district Faisalabad, Punjab, Pakistan. The study protocol was approved by the Institutional Review Board (IRB), Government College University, and Faisalabad, Pakistan (i.e., Ref.No. GCUF/ERC/2270). Furthermore, the protocol was also registered and approved by the Thai Clinical Trial Registry (i.e., TCTR20210703002 on 03 July, 2021) with the following URL: https://www.thaiclinicaltrials.org/show/TCTR20210703002).

### Participants

**Fig. 1:**
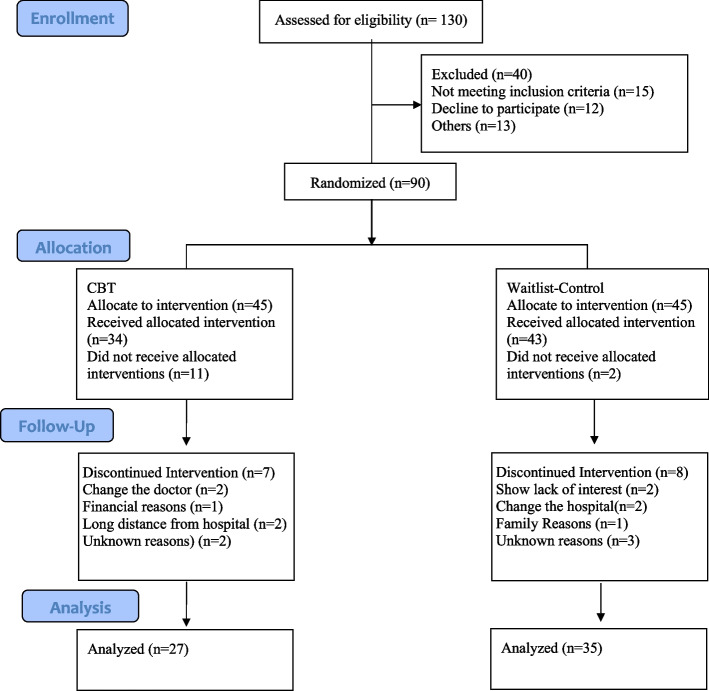
Flow Diagram of Patients with Type II Diabetes Mellitus

Consultant medical doctors first diagnosed patients with T2DM after evaluating them using medical evaluations from reliable laboratories (i.e., Agha Khan University Laboratories & Shaukat Khanum Memorial Trust Laboratories) at outpatient settings of different hospitals. and. Then the diagnosed participants were referred to us for the inclusion in our study. These participants first went through our clinical psychologists for psychological assessment and evaluation. The number of needed participants was calculated through G-Power software using the effect size (f) = 0.20, α = 0.05, power (1-β error prob.) =0.95 with actual power = 0.96 which gave us the total required sample size of56 (experimental and waitlist control conditions combined) [27]. Therefore, in this RCT, 130 participants were initially enrolled for eligibility assessment, 90 patients qualified the inclusion criteria, and they were allocated to two different treatment conditions (i.e., EXP = 45 & WLC = 45) through random assignment (see Fig. 1). A total of 62 patients completed all the required procedures of the present study. Participants’ age range was 23 to 50 years.

### Inclusion and exclusion criteria

In this RCT, people diagnosed with Type-II Diabetes Mellitus (T2DM) availing outpatient treatment facilities under consultant practitioners in different hospitals of Faisalabad were recruited. Participants who achieved mean item score of 3 or above (moderate distress) on DDS and were belonging to mild depressive symptoms category or higher on PHQ were included and allocated for two treatment conditions. Only those patients were included who had been diagnosed at-least 6-month ago (and at most 10 years ago) with T2DM and had been experiencing depressive symptoms for at-least two-weeks. Respondents who were suffering from major depressive disorder, persistent mood disorder and health anxiety disorder were excluded from the study as the protocol of their treatment was out of the scope of the present study. Patients were taken from all socioeconomic statuses. Participants with Type-I diabetes mellitus, duration of illness of less than 6 months and more than 10 years, not availing any medical treatment or receiving inpatient treatment were excluded from the study. Participants with any physical disability, severe physical/head injury, or undergoing any surgical treatment were also excluded from the study. Participants who did not sign the consent form or did not complete all the research procedures were also excluded from the study.

### Assessment and screening

A clinical psychologists conducted in-depth clinical interviews with patients in one-on-one settings. In the interview, the central focus was given to exploring the current presenting complaints, duration of illness, nature of the problems, and symptoms severity in terms of psychiatric problems. In order to cross check, the symptoms severity baseline measures, i.e., DDS (for diabetes distress) and PHQ (for depressive symptoms severity) were administered. Moreover, patients under treatment and experiencing T2DM for the last 6 months and experiencing depressive symptoms more than 2 weeks were selected for this RCT. Structured Clinical Interview for Diagnosis (SCID) was used to screen out any patients suffering from major depressive disorder, persistent mood disorders and health anxiety disorder and such patients were excluded. They were excluded from this RCT because they were referred for further psychological evaluation and treatment (i.e., psychiatric medication if need and extensive psychotherapy along with T2DM treatment). Furthermore, participants who achieved mean item score of 3 or above (moderate distress) on DDS and were belonging to mild depressive symptoms category or higher on PHQ were considered eligible for this RCT because they had diabetes distress and depressive symptoms due to T2DM and they were coming for treatment on regular bases. They were then randomly assigned to the two different treatment conditions.

### Procedure

Participant enrollment started on the 10th of July 2021, and trial completed on the 31st of December 2021, at different hospitals in the Faisalabad. Initially, participants’ baseline assessment was completed in the hospital’s outpatient setting. After qualifying the eligibility criteria, participants were interviewed and were invited for intake at the psychoogical clinic. Participants who met the study criteria and gave written informed consent to participate in the study were registered for this randomized clinical trial. Participants assigned to treatment conditions were assessed (pre-assessment), and at treatment completion, they were again assessed (post-assessment) with the duration of 4 months between the two assessments. We provided 8 to 10 CBT-based therapeutic sessions based on a particular agenda and goal (see interventions). Participants allocated to waitlist control were also assessed at pre-and post-assessment stages with the same time interval allocated for the experimental group.

### Randomization

We randomly assigned participants to treatment conditions so that both conditions were with similar and matchable group characteristics. Furthermore, all the patients were blinded with respect to the identification of the group they were assigned to. We told the participants that they were being randomized to receive psychological treatment, and its goal was to reduce their level of distress and depressive symptoms.

### Interventions

During the initial briefing, the participants were guided about the advantages of adherence and disadvantages of non-adherence, the importance of quality of life [23], reduced depression and anxiety [28], and also about improving their problem-solving abilities [29] to improve physiological functioning and to reduce stress and mood disturbance [30]. Then CBT was administered. CBT is an evidence-based intervention designed to address cognitive and behavioral problems efficiently. The CBT-based treatment plan was structured and delivered individually to each patient. Treatment was completed in 16 weeks, and therapeutic session frequency was one session in 10–12 days intervals with 45–60 minutes. In the initial sessions of the treatment, the therapist discussed about the process of treatment sessions, frequency, duration, the role of the therapist and client, the significance of patients’ active participation, and homework assignments. CBT protocol was structured according to Beck [31], Hilliard et al. [32] and Hood et al. [33]. The main components of the CBT were psychoeducation, cognitive conceptualization, adherence training, activity scheduling, problem-solving, improving coping strategies, muscle relaxation and imagery, and, lapse and relapse prevention (see supplementary material). The therapist asked the patients to come with a diary to write down important notes, daily homework assignments, and activity schedules and to write the current issues for discussion in the upcoming session. We received written feedback after each session. Almost all the patients easily understood the therapeutic process, therapy concept, content, and the mechanism for change.

#### Waitlist control condition

Participants allocated to the waitlist control condition received no treatment for 16 weeks. Their pre-and post-assessment was completed with the same time interval as that of the experimental group.

### Assessment measures

#### Demographic information

The demographic form that was used comprised of personal information from patients; such as age, education, socioeconomic status, family system, total family members, marital status, duration of illness, and the hospital name. In addition, the information related to their illness such as glucose level, duration of treatment, type of treatment, the dose of insulin, hypertension, obesity family history, was also asked in the demographic form. This form was used for further analysis and to tabulate results.

#### Primary outcomes measures

##### Diabetes distress scale (DDS-17) [34]

Diabetes Distress Scale DDS-17 is an instrument used to assess the level of distress among diabetes patients. It consists of 17 items. It has a six-point Likert scale where 1 means no problem and 6 indicates serious issues or problems. It has four subscales emotional-related distress, physician-related distress, regimen-related distress, and interpersonal-related distress. A mean item score of 3 or above is considered worthy of clinical attention (moderate distress). Its reliability was reported at 0.87.

##### The patient health questionnaire-9 (PHQ) [35]

The PHQ is a nine-item depression scale. It is based directly on the diagnostic criteria for major depressive disorders in the Diagnostic and Statistical Manual Fifth Edition. The PHQ scores range from 0 to 3 from not at all to nearly every day, respectively. It has five ranges of severity, i.e., minimal depressive symptoms, mild depressive symptoms, moderate depressive symptoms, moderately severe depressive symptoms, and severe depressive symptoms. The scores on this self-report measure do not provide a diagnosis on their own as a diagnosis necessitates a detailed clinical investigation. PHQ reliability estimation is 0.89.

#### Secondary outcomes measures

##### Short health anxiety inventory (SHAI) [36]

The SHAI is an instrument widely used to assess anxiety about the health status of a person. It consists of 18 items. Items assess the worries about health, awareness of bodily sensations or changes, and feared consequences of having an illness. The SHAI has demonstrated good reliability 0.86 and criterion validity 0.8.

##### Revised version of diabetes quality of life questionnaire (RV-DQOL) [37]

The RV-DQOL instrument is used to measure the quality of life among diabetes patients. It consists of 13 items and has three domains: satisfaction, impact, and worry. It has a 5-point Likert scale from “no impact/no worries” to “always satisfied/always affected.” The reliability of DQOL is 0.92 and 0.84, for worry, 0.98 and 0.60, for faction and, for “impact,” 0.99 and 0.57, respectively.

##### General medication adherence scale (GMAS) [38]

*The GMAS* is widely used to determine adherence among patients with chronic illness and is used for diabetes patients. This scale has three subscales; and each subscale measures a specific dimension of non-adherence., patient behavior-related non-adherence, additional diseases and pill burden-related non-adherence, and, cost-related non-adherence. It measures the overall adherence to medication as well. It has reliability estimation of 0.84, and test-re-test reliability of 0.99 and content validity of 0.80.

##### International physical activity questionnaire (IPAQ) [39]

The IPAQ measures the amount of physical activity performed over the past 7-day period. The IPAQ includes questions about the time spent engaging in vigorous physical activities, moderate physical activities, and walking. The IPAQ is a reliable (*p* = 0.76) and valid measure (concurrent = 0.58; criterion = 0.30).

### Statistical analysis

Descriptive statistics (Mean & SD) was used to calculate sample demographic characteristics, whereas, group characteristics were compared at pre-test using χ^2^ and *t*-test to compare variables. A repeated measures ANOVA statistic was used for the assessment time (pre- versus post-test) to evaluate the effects and benefits of the interventions (within-group effects). Frequency distribution statistics were used to find out the severity of the symptoms. An alpha of .05 was used for all analyses, and *p-*value <.01was submitted to Bonferroni correction using IBM SPSS Statistics (Version 25).

## Results

### Recruitment and attrition

A total of 130 participants were recruited, and *n* = 90 met the inclusion criteria. Participants were equally divided into experimental (EXP) = 45(50%) and waitlist control (WLC) = 45(50%), and they were statistically analyzed (see Fig. 1). There were no significant differences found between EXP vs. WLC among the demographic characteristics; such as age, gender, education, glucose level, family system, occupation, socioeconomic status, duration of illness, types of treatment, duration of treatment, checkup, hypertension, BMI, family history and obesity respectively (see Table 1).

**Table 1:** Comparison of participants’ demographic characteristics groups wise and overall

*Variables*	*Category*	*N*	*Groups*			
*Characteristics*			*Experimental*	*Control*	*X*^*2*^*/t*	*P*
*N Targeted*		130				
*N Allocated, n*		90	45	45		
*N final* n(%)		62	27(33.30%)	35(43.20%)		
Age, M (SD)		90	36.93(6.87)	37.62(6.77)	−.48	.64
Gender	Female n(%)	50	23(51.20%)	27(60.10%)	.72	.40
	Male n(%)	40	22(48.90%)	18(40.00%)		
Education	<Matric n(%)	35	17(37.00%)	18(33.30%)	1.09	.28
	Matric n(%)	50	26(57.70%)	24(45.30%)		
	Above n(%)	05	02(4.30%)	03(4.30%)		
Glucose Level	< 250 n(%)	12	08(17.70%)	04(8.80%)	−1.12	.27
	> 250–350 n(%)	48	19(42.20%)	29(64.50%)		
	> 350–500 n(%)	30	18(40.00%)	12(26.70%)		
Family System	Nuclear n(%)	57	31(68.90%)	26(57.70%)	1.20	.28
	Joint n(%)	33	14(31.20%)	19(42.30%)		
Occupation	Unemployed n(%)	48	21(46.70%)	27(60.00%)	1.61	.21
	Employee n(%)	42	24(53.40%)	18(40.00%)		
SES	Low n(%)	51	24(53.30%)	27(60.10%)	.62	.74
	Middle n(%)	29	15(33.30%)	14(31.20%)		
	High n(%)	10	6(13.30%)	04(8.80%)		
Duration. of. Illness	> 2 years n(%)	28	12(26.60%)	16(35.50%)	4.31	.23
	3–5 years n(%)	40	18(40.00%)	22(48.90%)		
	5–10 years n(%)	21	14(31.20%)	07(15.50%)		
Type of Treatment	Insulin n(%)	41	22(48.80%)	19(42.30%)	.75	.69
	Medication n(%)	42	19(42.30%)	23(51.20%)		
	Both n(%)	07	04(8.80%)	03(6.60%)		
Duration of Treatment	> 6-12 months n(%)	38	19(42.30%)	19(42.30%)	.72	.70
	2–3 years n(%)	31	14(31.10%)	17(37.80%)		
	4–6 years n(%)	21	12(26.60%)	09(20.00%)		
Check-up	Daily n(%)	37	19(42.30%)	18(40.10%)	.96	.62
	Weekly n(%)	28	16(35.60%)	12(26.70%)		
	Monthly n(%)	25	14(31.20%)	11(24.50%)		
Hypertension	yes n(%)	58	27(60.10%)	31(68.90%)	.78	.38
	No n(%)	32	18(40.10%)	14(31.10%)		
BMI	Healthy n(%)	26	12(26.60%)	14(31.10%)	.54	.77
	Overweight n(%)	25	14(31.10%)	11(24.50%)		
	Obese n(%)	39	19(42.20%)	20(44.50%)		
Family History	Present n(%)	56	26(57.80%)	30(66.70%)	.76	.39
	Not Present n(%)	34	19(42.20%)	15(33.40%)		
Obesity	Yes n(%)	59	31(68.90%)	28(62.30%)	.45	.51
	No n(%)	31	14(31.20%)	17(37.80%)		
PHQ		90	16.18(3.85)	15.73(3.83)	.05	.96
SHAI		90	30.11(6.55)	30.02(7.16)	.18	.86
EBS		90	3.70(0.86)	3.77(0.74)	−.37	.72
PDS		90	4.85(0.75)	4.17(0.80)	.89	.22
RDS		90	4.65(0.76)	4.13(0.82)	.75	.35
IDS		90	3.52(1.14)	3.82(0.86)	−.76	.45
DDS		90	16.45(2.73)	15.90(2.76)	.54	.68
PBNA		90	9.11(2.51)	9.49(2.55)	−.08	.99
ADPB		90	7.76(2.14)	7.53(2.19)	− 1.20	.23
CRNA		90	3.98(1.43)	3.76(1.30)	.53	.59
GMAS		90	20.84(4.82)	20.78(5.00)	−.46	.65
SATS		90	67.11(13.53)	70.30(13.74)	−.39	.70
IMPS		90	69.89(12.32)	74.11(12.94)	−1.35	.18
WORS		90	64.89(16.11)	69.19(18.11)	−.80	.42
DQLS		90	67.45(10.58)	71.21(12.12)	−.94	.35
WALS		90	88.24(14.35)	87.80(15.20)	−1.33	.19
MPAS		90	78.00(12.00)	79.00(13.10)	1.03	.31
VPAS		90	65.35(12.36)	64.95(11.93)	−1.02	.32
IPAQ		90	98.26(33.10)	96.56(34.25)	−.93	.36

### Primary and secondary outcomes

We found a significant mean difference between EXP and WLC conditions in post-testing scores on PHQ which indicates CBT substantially decreased depressive symptoms among patients with T2DM. Findings indicate that significant mean differences were found between EXP and WLC groups on SHAI which indicates that the CBT played a 77% role in reducing the level of health anxiety in the experimental group. Furthermore, significant mean differences were found between baseline and post-testing scores on the scale of EBS, PDS, RDS, IDS and overall DDS between EXP and WLC which indicates CBT reduced 63% emotional burden, 68% physician burden, 66% of regimen distress, 53% of interpersonal distress and 76% of overall diabetes distress of EXP group. Similarly, EXP group was found to be significantly different as compared to WLC on PBNA, ADPB, CRNA and overall GMAS which shows that CBT also improved adherence to treatment; such as: 48% patient behavior related non-adherence, 42% additional disease and pill burden describe non-adherence, 51% cost related non adherence and 57% over all general medication adherence to treatment. Analysis reveals a significant difference between EXP and WLC after CBT on SATS, IMPS, WORS, and overall DQLS which shows CBT sessions improved satisfaction to the degree of 41%, impact to the degree of 43%, non-worried behavior to the degree of 41%, and, overall diabetes patients’ quality of life to the degree of 58%. Furthermore, findings show that CBT produced a significant difference in changing lifestyle as compared to WLC on WALS, MPAS, VPAS and IPAQ which shows that patients with T2DM improved their quality of life after getting CBT sessions; such as: walking activities to the degree of 69%, moderate physical activities to the degree of 23%, vigorous physical activities to the degree of 33%, and overall daily physical activities to the degree of 73% (see Table 2).

**Table 2:** Mean (standard deviation) and repeated-measure ANOVA of clinical scores during pre- and post-test interventions

Groups						
	**Experimental**		**Waitlist-Control**			
	Baseline	Post-Test	Baseline	Post-Test		
	M (SD)	M (SD)	M (SD)	M (SD)	F	η^2^
PHQ	15.85(3.81)	8.81(2.59)	15.80(4.12)	16.54(3.97)	94.44***	.62
SHAI	29.81(6.83)	15.26(1.94)	29.49(7.17)	32.29(6.04)	201.92***	.77
EBS	3.72(0.90)	2.36(0.55)	3.79(0.75)	3.93(0.78)	103.64***	.64
PDS	4.57(0.73)	2.67(0.73)	4.10(0.86)	4.04(0.78)	129.09***	.68
RDS	4.60(0.72)	2.71(0.689)	4.12(.88)	3.85(0.76)	118.63***	.67
IDS	3.56(1.12)	2.31(0.62)	3.75(.93)	3.61(0.87)	29.71***	.31
DDS	16.46(2.71)	10.05(2.22)	15.77(2.99)	15.45(2.93)	222.71***	.79
PBNA	8.78(2.34)	28.19(1.41)	9.51(2.39)	9.77(1.99)	56.33***	.49
ADPB	7.48(2.19)	10.93(1.33)	7.49(2.06)	7.29(1.78)	43.65***	.42
CRNA	3.96(1.51)	5.89(0.32)	3.77(1.301)	3.37(1.26)	61.65***	.51
GMAS	20.22(4.73)	28.19(3.04)	20.77(4.61)	20.86(3.80)	67.58***	.57
SATS	67.65(13.20)	49.51(11.76)	69.05(14.38)	69.71(11.45)	41.37***	.41
IMPS	68.89(12.28)	47.04(11.29)	73.43(13.66)	71.71(9.85)	44.13***	.43
WORS	64.69(15.89)	45.93(13.76)	68.19(17.79)	72(14.36)	40.86***	.41
DQLS	67.35(10.41)	12.85(9.99)	70.20(12.79)	70.86(9.85)	83.35***	.58
WALS	389.27(323.7)	1103.6(260.90)	550.3(505.5)	550.3(505.5)	134.70***	.70
MPAS	171.1(286.20)	456.2(453.80)	84.0(240.50)	96.0(271.10)	17.61***	.28
VPAS	41.48(149.45)	528.88(598.18)	96.0(395.60)	96.0(395.60)	29.74***	.33
IPAQ	601.8(512.84)	20.88(925.4)	735.6(861.8)	747.97(889.88)	164.25***	.74

Note: *** = *p* < .001; η^2^ = Partial Square Eta; *PHQ* Patients Health Questionnaire, *SHAI* Short Health Anxiety Inventory, *EBS* Emotional Burden Subscale, *PDS* Physician Distress Subscale, *RDS* Regimen Distress Subscale, *IDS* Interpersonal Distress Subscale, *DDS* Diabetes Distress Scale, *PBNA* Patient Behavior related Non-Adherence, *ADPB* Additional Disease and Pill Burden Related Non-Adherence, *CRNA* Cost Related Non-Adherence, *GMAS* General Medication Adherence Scale, *SATS* Satisfaction Subscale, *IMPS* Impact Subscale, *WORS* Worry Subscale, *DQLSL* Diabetes Quality of Life Scale, *WALS* Walking Subscale, *MPAS* Moderate Physical Activity Subscale, *VPAS* Vigorous Physical Activity Subscale, *IPAQ* International Physical Activity Questionnaire

**Fig. 2:**
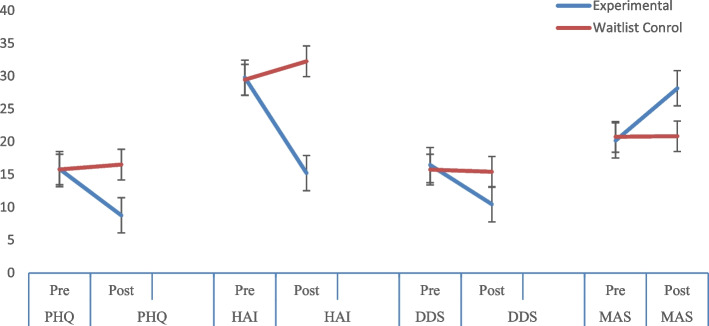
Symptomatic change in PHQ, HAI, DDS and MAS scores over the course of treatment between experimental and waitlist control groups. *Note: PHQ = Patients Health Questionnaire; HAI = Health Anxiety Inventory; DDS = Diabetes Distress Scale; MAS = Medical Adherence Scale*

The analysis reveals that scores significantly decreased throughout the treatment among the experimental group on PHQ, HAI, and DDS, and the group’s scores enhanced on MAS. CBT played an influential role in addressing depressive symptoms, health-related anxiety, and diabetes distress. On the other hand, CBT played a supportive role in increasing treatment adherence (see Fig. 2).

Findings showed a significant improvement in symptoms of depression in the EXP group. The score in the pre-vs. post of the EXP group is as follows: minimal depressive symptoms 0% vs. 3.7%, mild depressive symptoms 7.4% vs.37.1%, moderate depressive symptoms 40.8% vs. 59.3%; this indicates that CBT significantly reduced depressive symptoms. Similarly, at pre-assessment, the moderate-severe depressive symptoms were in 33.4% of the participants, and severe depressive symptoms were in 18.5% of the participants which got reduced due to CBT at post-assessment, while no significant change was observed in the waitlist control group (see Table 3).

**Table 3:** Range of symptoms severity on Patients Health Questionnaire (PHQ) between experimental and waitlist control groups at pre-and post-assessment scores

Groups	Severity level	PHQ	
		Pre-Scores	Post-Scores
Experimental Group (EXP = 27)		n(%)	n(%)
	Minimal depressive symptoms	–	1(3.7%)
	Mild depressive symptoms	2(7.40%)	10(37.1%)
	Moderate depressive symptoms	11(40.8%)	16(59.3%)
	Moderate to severe depressive symptoms	9(33.4%)	–
	Severe depressive symptoms	5(18.5%)	–
Waitlist Control Group (WLC = 35)			
	Minimal depressive symptoms	–	–
	Mild depressive symptoms	2(5.7%)	1(2.9%)
	Moderate depressive symptoms	9(25.7%)	9(25.7%)
	Moderate to severe depressive symptoms	18(51.4%)	17(48.8%)
	Severe depressive symptoms	6(17.2%)	8(22.8%)

Findings show that CBT significantly improved adherence to treatment among individuals with T2DM. Post-testing analysis reveals that the individuals from the EXP group got an improvement in their treatment adherence considerably. For example, high adherence, which was at 2(7.41%) at baseline, increased 10(37.04%), good adherence, which was at 3(11.12%) at baseline, improved to 12(44.45%), partial adherence, which was at 13(46.15%) at the baseline, shrank to 3(11.12%) as people moved to higher categories, and, low adherence, which was at 6(22.23%) at baseline, again shrank to 2(7.41%) as people moved to higher categories. In the case of WLC, no significant difference was observed, such as, high adherence at baseline had 3(8.57%) participants and at post-analysis stage there were 2(5.71%); good adherence at baseline had 6(17.14%) participants and at post-analysis stage there were 5(14.29%); partial adherence at baseline had 15(42.86%) participants and at post-analysis stage there were 18(51.43%); low adherence at baseline had 9(25.71%) participants and at post-analysis stage there were 6(17.14%); whereas, poor adherence at baseline had 2(5.71%) participants and at post-analysis stage there were 4(11.43%) (see Table 4).

**Table 4:** Score difference on General Medical Adherence Scale (GMAS) between experimental and waitlist control groups at pre-and post-assessment scores

Groups	Levels	GMAS	
		Pre-Scores	Post-Scores
Experimental Group (EXP = 27)		n(%)	n(%)
	High Adherence	2(7.41%)	10(37.04%)
	Good Adherence	3(11.12%)	12(44.45%)
	Partial Adherence	13(48.15%)	3(11.12%)
	Low Adherence	6(22.23%)	2(7.41%)
	Poor Adherence	3(11.12%)	–
Waitlist-Control Group (WLC = 35)			
	High Adherence	3(8.57%)	2(5.71%)
	Good Adherence	6(17.14%)	5(14.29%)
	Partial Adherence	15(42.86%)	18(51.43%)
	Low Adherence	9(25.71%)	6(17.14%)
	Poor Adherence	2(5.71%)	4(11.43%)

## Discussion

Our findings present the effectiveness of CBT for patients with T2DM in order to produce substantial improvement on multiple psychophysiological health outcomes. CBT has been widely used to treat patients’ psychological problems with diabetes [23]. Overall findings of our study show substantial improvement in diabetes distress, health anxiety, depression, quality of life, and medication adherence among the experimental group. Whereas, no difference was found in the pre- and post-testing in the control group.

Our findings show that CBT effectively addresses patients’ psychological problems with diabetes mellitus. These findings are consistent with the findings of the previous studies. CBT-based interventions help patients by bringing about psycho-education, development of better understanding and upping the motivation to of overcome and manage negative automatic thoughts, by regulating emotions, and rectifying negative beliefs [40, 41]. In our findings, CBT effectively reduced psychological distress and improved emotional and behavioral outcomes as well as medication adherence [42]. Significant difference between baseline and outcomes assessment scores in the experimental group report that CBT was found an evidence-based treatment intervention to reduce diabetes distress and depressive symptoms among patients with T2DM [40].

Moreover, the analysis reveals that CBT effectively helped the patients to develop a positive attitude toward life and promoted their functional outcomes through skill training [43, 44]. This reflects that CBT is an effective intervention to address depressive symptoms among T2DM [41]. Depression is a ubiquitous affliction that affects patients with diabetes. That’s why it is necessary to encounter this, and for this purpose, CBT is a strong and reliable treatment option to follow [43].

Furthermore, our analysis reveals that CBT significantly decreases health anxiety among T2DM patients in our study [45]. Health anxiety is common among patients with diabetes. Patients with DM have a propensity for developing apprehension about their illness, that’s why it was reported in our sample that after receiving the intervention, participants’ level of anxiety was reduced [46]. This maybe so because the intervention was aimed to change the thought pattern into adaptive thinking pattern in T2DM patients.

In our study. CBT was tested to improve the quality of life among patients with T2DM because diabetes negatively affects quality of life [47]. There was a significant difference between pre- and post- test scores; participants in the experimental group showed higher quality of life. The experimental group’s post-testing was remarkably different from the control group as their quality of life improved after the intervention. However, the control group didn’t significantly differ because they didn’t receive the intervention [48].

The study findings also indicate that CBT improved medication adherence. Medication adherence was focused upon because it is strongly associated with many complications of T2DM [49]. In our study, medication adherence was low at baseline screening because many patients were not taking medication appropriately. They were less likely to seek medication and held an unhealthy diet plan that led to poor glycmeic control. Hence, we used CBT to manage medication adherence to overcome this issue of the patients with T2DM, and substantial improvement was reported in the experimental group. However, no difference was found in the control group.

Our findings indicate that patients with diabetes mellitus showed more physical activity after having CBT; these findings are consistent with the existing research [26]. Physical activity accounts for maximum improvement in diabetic patients because it enhances self-care behavior, diminishes glucose levels, and improves health status. It is important to address the physical activity among T2DM to improve their lifestyle and to balance their glucose and insulin levels to prevent them from facing damaging health outcomes. For this, researchers can use CBT and other different motivational programs to reduce the level of inactivity among diabetes patients [50].

## Conclusion

It is concluded that CBT produced substantial improvement among patients with T2DM having diabetes distress and depressive symptoms. It has also been supported that CBT is an effective and evidence-based treatment to address diabetes distress, depressive symptoms, and health-related anxiety. CBT also improves treatment adherence and quality of life among diabetic patients. This also helps the patients to manage daily routines, sustain motivation for treatment and develop a positive attitude toward life. Finally, CBT increases treatment adherence in patients with T2DM helping being about a rapid recovery process.

### Limitations of the study

The current study has some limitations. First, only patients with Type II diabetes mellitus were taken in the study. Second, patients were taken only from outpatient departments. Third, major focus was given only to diabetes distress, depressive symptoms, health anxiety, medication adherence and physical activities; many other factors; such as, patients’ coping mechanisms, social and emotional support systems, and others comorbid medical conditions, diagnosed psychiatric disorders and aging factors, were not investigated in this study. Fourth, the cognitive behavior therapy session was mainly designed for patients with T2DM having psychiatric problems, not psychiatric disorders. Fifth point I that we could not enroll a large-enough number of participants (due to Covid-19) in this RCT that come from different age groups, especially above age 50, who might have more diabetes problems. Lastly, this RCT does not say anything about the patients who have a history of chronic diabetes greater than 10 years. Nonetheless, if future researches target the aforesaid areas, it will be a helpful contribution to the literature as well as to the generalizability of the results.

### Recommendation and implication of the study

This paper provides contributions to fill the knowledge gap in this ignored area of research. This paper also recommends that future researchers should check the effectiveness of CBT for patients with T1DM and T2DM having severe depressive and anxiety-related disorders. This study provides valuable background for mental health practitioners to treat and develop the guidelines and protocol for patients with diabetes mellitus and other chronic illnesses such as diabetes, cancer, HIV/AIDS. Cognitive behavior therapy played a substantial role in developing patient insight and motivation, so, it is also recommended that other practitioners should carry out qualitative studies on the variables of this research in order to know the qualities and limitations of this strategy and give even more suitable strategies to deal with the problems faced with T2DM within the field of cognitive behavior therapy.

## Supplementary Information

**Additional file 1: Supplementary Table 1.** Therapeutic sessions details with designed agenda and content for the patients withT2DM.

## Data Availability

The dataset generated and/or analyzed during the present study are not publicly available because no permission was taken from the participants and the hospital administration where the study was conducted. The datasets are available from the corresponding authors on a special request.

## Abbreviations

CBT: Cognitive Behavior Therapy
T2DM: Type 2 Diabetes Mellitus
PHQ: Patient Health Questionnaire-9
SHAI: Short Health Anxiety Inventory
DD: Diabetes Distress
HA: Health Anxiety
QOL: Quality of Life
EXP: Experimental group
WLC: Waitlist Control
EBS: Emotional Burden Subscale
PDS: Physician Distress Subscale
RDS: Regimen Distress Subscale
IDS: Interpersonal Distress Subscale
DDS: Diabetes Distress Scale
PBNA: Patient Behavior related Non-Adherence
ADPB: Additional Disease and Pill Burden Related Non-Adherence
CRNA: Cost Related Non-Adherence
GMAS: General Medication Adherence Scale
SATS: Satisfaction Subscale
IMPS: Impact Subscale
WORS: Worry Subscale
DQLSL: Diabetes Quality of Life Scale
WALS: Walking Subscale
MPAS: Moderate Physical Activity Subscale
VPAS: Vigorous Physical Activity Subscale
IPAQ: International Physical Activity Questionnaire.
