# Supplementary material for: Cognitive behavior therapy for diabetes distress, depression, health anxiety, quality of life and treatment adherence among patients with type-II diabetes mellitus: a randomized control trial
Source: BMC Psychiatry. 2023 Feb 3;23:86. doi: 10.1186/s12888-023-04546-w (PMC9896442; doi:10.1186/s12888-023-04546-w)
Supplement: Supplementary file 1 — Additional file 1: Supplementary Table 1. Therapeutic sessions details with designed agenda and content for the patients withT2DM. [file 12888_2023_4546_MOESM1_ESM.docx]

Therapeutic sessions details with designed agenda and content for the patients withT2DM

| Cognitive Behavior Therapy Program for Type II Diabetes Patients | | |
| --- | --- | --- |
| N.O.S | Agenda | Content |
| 1 | Psycho-education | To provide feedback to the patients and educate them on how to improve treatment adherence, how diabetes distress leads to depression and anxiety-related symptoms, and how these problems affect their quality of life (Hati et al., 2020). |
| 2 | Cognitive conceptualization | Identification of distortions. Identification of negative schemas, thoughts, feelings, and emotions, and irrational and inflexible beliefs about diabetes. Evaluate and respond to the automatic thoughts and negative core beliefs about diabetes—implementing the ABC model. Cognitive restructuring. Use of alternative ideas and beliefs ([Hamiel](javascript:;)  2020). |
| 3 | Adherence training | Educate about treatment adherence. Educate to cope with the side effects of medications and medical regimens. Formulate a daily schedule for Medicine and other self-care behaviors. Educate about treatment adherence. Develop cues for taking medication or implementing other self-care procedures (i.e., glucose monitoring) (Andreae et al., 2020; Kretchy et al., 2020). |
| 4 | Activity Scheduling | Monitor and modify the activities of the client daily. Increasing activities of pleasure or mastery and reducing inactivity Focus on maintaining healthy social interaction and engaging in leisure activities. Reduce Poor self-care behaviors CBT-based intervention improves functioning, QOL, and self-reported physical activity (Andreae et al., 2020). |
| 5 | Problem Solving | To identify the problem that leads to diabetes distress, depression, and health-related anxiety and list all possible solutions. Prioritizing solutions. List the pros and cons of each solution. Review the whole list, and give a rating to each solution. Implement the Best Option It is essential to address problem-solving among diabetes patients (ji et al., 2021). |
| 6 | Improving coping abilities | Cognitive reappraisal to develop healthy coping strategies among patients. Healthy coping with monitoring activities and behavior patterns associated with pleasure and positive emotions To improve strategies for coping with distress to reduce depressive and anxiety-related symptoms (Knowles et al., 2020) |
| 7 | Muscle relaxation & breathing exercise | Management of diabetes distress, depression, and anxiety Coping with physiological symptoms of distress Improved physiological symptoms related to distress.Reduce level o stress and improve Mood disturbance; self-help training to reduce stress and awareness to differentiate between healthy and unhealthy stress (Izgu et al., 2020) |
| 8 | Lapse &relapse prevention | Educate the client on how they can control, manage and regulate the triggers, situations, and incidences if they happen in the future (Lawlor et al., 2020) |
